# Social determinants of health: does socioeconomic status affect access to staging imaging for men with prostate cancer

**DOI:** 10.1038/s41391-022-00508-7

**Published:** 2022-02-15

**Authors:** Brian D. Kelly, Marlon Perera, Damien M. Bolton, Nathan Papa

**Affiliations:** 1 Department of Urology, Austin Health, Heidelberg, Melbourne, VIC Australia; 2 Department of Surgical Oncology, Peter MacCallum Cancer Centre, Melbourne, VIC Australia; 3 Department of Urology, Memorial Sloan Kettering Cancer Center, NYC, NY USA; 4 School of Public Health and Preventive Medicine, Monash University, Melbourne, VIC Australia

**Keywords:** Cancer epidemiology, Prostate cancer, Prostate cancer

## Abstract

Socioeconomic status (SES), race and geographical factors are known to impact prostate cancer management and outcomes. We aimed to assess these factors with regard to access to novel imaging in prostate cancer. Using the Prostate Cancer Outcomes Registry of Victoria (PCOR-Vic) we identified 5256 men diagnosed with prostate cancer via biopsy. Following the introduction of government rebate, the access to MRI improved with respect to SES. Access to PET imaging remains poor with respect to SES and geographical location in the absence of Federal funding. Further improvements for men with low SES and regional areas to access PET staging.

Social determinants of health (SDOH) include socioeconomic status (SES), race and geographical access to healthcare. Low SES is associated with a higher incidence of higher-grade prostate cancer at diagnosis and increased mortality, the cause of which is multifactorial and complex [1–4]. However, it is likely that access to key diagnostic modalities are implicated. In prostate cancer, significant advances to radiologic staging have occurred following the introduction of multiparametric magnetic resonance imaging (MRI) [5] and positron emission tomography (PET) [6, 7]. We aimed to assess the impact of SDOH on access to novel imaging modalities in prostate cancer.

It is pertinent to note that to improve prostate cancer management in men in Australia, mpMRI of the prostate for patients suspected of having prostate cancer has been subsidised by the Australian government in July 2018. This has enabled greater access for the general public via the public healthcare system, as patients meeting the Medicare Benefits Scheme (MBS) criteria can undergo mpMRI with no out-of-pocket fee [8]. Once a service is added to the Medicare benefit schedule, all men have access to the rebated service, regardless of income or SES status. At present, with current applications pending, PET imaging has not received similar federal subsidisation.

We performed a review of the Prostate Cancer Outcomes Registry of Victoria (PCOR-Vic) in July 2020 assessing the SDOH of men newly diagnosed with prostate cancer from January 2017 to December 2018 [9]. The registry does not collect ethnicity but does record Australian Aboriginal or Torres Strait Islander status. These men comprised <0.2% of the cohort, the small numbers precluded meaningful analysis. Using Australian Bureau of Statistics correspondences and linked geocoded data from the Victorian Cancer Registry, we assigned each deidentified patient a percentile rank of SES and determined their major metropolitan or regional residence status. The measure of SES used in this study is the Index of Relative Socio-economic Advantage and Disadvantage (IRSAD) published by the Australian Bureau of Statistics [10]. This measure incorporates numerous variables but the strongest indicators of low SES are an annual household income in the lowest two deciles (<A$26,000) and non-completion of the final year of high school. Locally weighted, running mean smoothing plots were generated to illustrate changes over time for the SDOH metrics in men receiving specific imaging compared to the whole sample population. For CT, bone scan and PET regressions, only men with grade group 2 and PSA > 10 ng/ml or grade group 3+ were analysed.

During this timeframe there were 5256 men diagnosed via prostate biopsy. Patient demographics and characteristics are summarized in Supplementary Table 1. Prior to the MBS reimbursement, the mean percentile difference in SES between patients receiving MRI or not was 5.7 (95% CI: 3.5–7.0) favouring more advantaged men. After July 2018, the difference was 3.5 (95% CI: 0.5–6.5). The running mean difference showed a narrowing of the SES access gap beginning prior to the MBS change (Fig. 1A). The SES gap for PET imaging was maintained over the 2 years at an average of 9.0 (95% CI: 6.2–1.7). No large percentage difference, 1.6 (95% CI: −1.1–4.4), was observed favouring metropolitan vs regional residence for access to MRI. For PET, the corresponding difference was 8.1 (95% CI: 4.5–11.6) with the regional access gap not appearing to decrease (Fig. 1B).

**Fig. 1:**
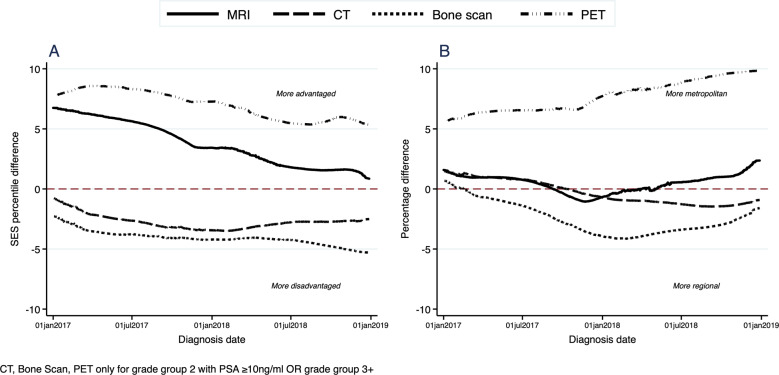
Locally weighted smoothed running mean differences between patients receiving the imaging modality and the total sample. **A** Difference in SES percentile. **B** Percentage difference between residence in major city and regional areas.

Our results suggest limited access to PET for prostate cancer in patients with lower SES. Conversely, access to MRI in patients with lower SES improved along with the introduction of federally subsidised MRI scans via the Medicare Benefits Scheme. It is likely that PET uptake is following a similar uptake pattern to that of MRI prior to subsidisation, implying that men with lower SES have reduced access to PET imaging due to significant out-of-pocket expenses. A subsequent analysis following the likely upcoming listing of PET imaging on the MBS will be of interest.

The current analysis highlights the compromised access to novel imaging modalities in patients who live outside of a major city. There is similar access to MRI, CT and bone scans for most men irrespective of their geographic location. However, over time, regional access to PET imaging has not improved. This perhaps is a reflection of the fact that most small regional medical centers do perform CT, MRI and bone scans whereas PET imaging is predominantly only available at large public and private institutions within metropolitan Melbourne. Further, men with low SES and living in regional Victoria are perhaps unwilling to travel to a large academic institution in the metropolitan city of Melbourne to access a PET scan or to enrol in a clinical trial assessing the use of various PET imaging.

Given the improvements in access to MRI for men of a low SES, which improves cancer detection of clinically significant cancer, there needs to be further improvements for men with low SES to access superior PET staging, and in particular for low SES men from regional areas.

## Supplementary information

Supplementary Table 1
