# Supplementary material for: Social determinants of health: does socioeconomic status affect access to staging imaging for men with prostate cancer
Source: Prostate Cancer Prostatic Dis. 2022 Feb 15;26(2):429–31. doi: 10.1038/s41391-022-00508-7 (PMC9376196; doi:10.1038/s41391-022-00508-7)
Supplement: Supplementary file 1 — Supplementary Table 1 [file 41391_2022_508_MOESM1_ESM.docx]

*Supplementary Table 1. Characteristics of the sample, N=5256*

| Age at diagnosis, years; median (IQR) | 67 (61 – 72) |
| --- | --- |
| Residence; n (%)  Metropolitan  Regional | 3657 (70)  1599 (30) |
| Socioeconomic quintile, n (%)  1^st^ (most disadvantaged)  2^nd^  3^rd^  4^th^  5^th^ (most advantaged)  Not available | 829 (16)  976 (19)  1038 (20)  1102 (21)  1298 (25)  13 (0.2) |
| Staging imaging, n (%)  MRI  CT  Bone scan  PET | 1971 (38)  2031 (39)  1922 (37)  812 (15) |
